# Effects of *HAR1* on cognitive function in mice and the regulatory network of *HAR1* determined by RNA sequencing and applied bioinformatics analysis

**DOI:** 10.3389/fgene.2023.947144

**Published:** 2023-03-08

**Authors:** Luting Zhang, Shengmou Lin, Kailing Huang, Allen Chen, Nan Li, Shuhan Shen, Zhouxia Zheng, Xiaoshun Shi, Jimei Sun, Jingyin Kong, Min Chen

**Affiliations:** ^1^ Department of Obstetrics and Gynecology, Department of Fetal Medicine and Prenatal Diagnosis, Key Laboratory for Major Obstetric Diseases of Guang-Dong Province, The Third Affiliated Hospital of Guangzhou Medical University, Guangzhou, China; ^2^ Department of Obstetrics and Gynecology, The University of Hong Kong—Shenzhen Hospital, Shenzhen, China; ^3^ The First School of Clinical Medicine, Southern Medical University, Guangzhou, China; ^4^ Guangzhou Mendel Genomics and Medical Technology Co., Ltd., Guangzhou, China; ^5^ Imperial College London, London, England

**Keywords:** *HAR1*, lncRNA, brain development, transgenic mouse, RNA-seq

## Abstract

**Background:** *HAR1* is a 118-bp segment that lies in a pair of novel non-coding RNA genes. It shows a dramatic accelerated change with an estimated 18 substitutions in the human lineage since the human–chimpanzee ancestor, compared with the expected 0.27 substitutions based on the slow rate of change in this region in other amniotes. Mutations of *HAR1* lead to a different HAR1 secondary structure in humans compared to that in chimpanzees.

**Methods:** We cloned *HAR1* into the EF-1α promoter vector to generate transgenic mice. Morris water maze tests and step-down passive avoidance tests were conducted to observe the changes in memory and cognitive abilities of mice. RNA-seq analysis was performed to identify differentially expressed genes (DEGs) between the experimental and control groups. Systematic bioinformatics analysis was used to confirm the pathways and functions that the DEGs were involved in.

**Results:** Memory and cognitive abilities of the transgenic mice were significantly improved. The results of Gene Ontology (GO) analysis showed that Neuron differentiation, Dentate gyrus development, Nervous system development, Cerebral cortex neuron differentiation, Cerebral cortex development, Cerebral cortex development and Neurogenesis are all significant GO terms related to brain development. The DEGs enriched in these terms included *Lhx2*, *Emx2*, *Foxg1*, *Nr2e1* and *Emx1*. All these genes play an important role in regulating the functioning of Cajal–Retzius cells (CRs). The DEGs were also enriched in glutamatergic synapses, synapses, memory, and the positive regulation of long-term synaptic potentiation. In addition, “cellular response to calcium ions” exhibited the second highest rich factor in the GO analysis. Kyoto Encyclopedia of Genes and Genomes (KEGG) analysis of the DEGs showed that the neuroactive ligand–receptor interaction pathway was the most significantly enriched pathway, and DEGs also notably enriched in neuroactive ligand–receptor interaction, axon guidance, and cholinergic synapses.

**Conclusion:** *HAR1* overexpression led to improvements in memory and cognitive abilities of the transgenic mice. The possible mechanism for this was that the long non-coding RNA (lncRNA) *HAR1A* affected brain development by regulating the function of CRs. Moreover, *HAR1A* may be involved in ligand–receptor interaction, axon guidance, and synapse formation, all of which are important in brain development and evolution. Furthermore, cellular response to calcium may play an important role in those processes.

## 1 Introduction

Human accelerated regions (HARs) consist of 49 segments of the human genome that have been conserved through vertebrate evolution, although they are strikingly different in humans (Hubisz and Pollard, 2014). HARs may be responsible for the unique characteristics of our species, since they are related to the evolution of size, structure, and complexity of the human brain. *HAR1* is a human genome region located in the long arm of chromosome 20. This 118-bp region contains 18 mutations between humans and chimpanzees. These mutations cause different secondary structures of lncRNA *HAR1A* between humans and chimpanzees (Pollard et al., 2006a). *HAR1A,* in particular, is one of the most distinctively different HARs between humans and chimpanzees (Pollard et al., 2006b; Kostka et al., 2012). Furthermore, dysregulation of *HAR1A* has been associated with many central nervous system diseases.

LncRNA *HAR1A* is explicitly expressed in Cajal–Retzius cells (CRs), which are well-known for controlling nerve cell migration during brain development, suggesting that *HAR1A* is important in brain development (Cao et al., 2021; Lee et al., 2021). In addition, the expression of lncRNA *HAR1A* affects the occurrence and development of many brain diseases, including Huntington’s disease and glioma (Chen et al., 2018; Wei et al., 2021). However, genes regulated by *HAR1A* in the developing brain have not been systematically studied yet. Therefore, its association with the development and evolution of the central nervous system remains relatively unclear.

*HAR1A* belongs to lncRNAs, which are non-coding RNAs longer than 200 nucleotides. Accumulating evidence has demonstrated that lncRNAs exert epigenetic effects by regulating transcriptional and post-transcriptional processes. Moreover, lncRNAs have been reported to regulate human brain development, neural stem cell regulation, and neuronal axon elongation (Chen et al., 2018; Wei et al., 2021). The involvement of lncRNAs in regulating neurological growth and development allows the nervous system to grow and differentiate in the regular order of time and space (Wei et al., 2021).

In this study, we explored the effects of *HAR1* overexpression on the cognition and memory of mice and analyzed the related functions and enriched pathways of differentially expressed genes (DEGs). This study aimed to identify the role of *HAR1* in brain development and deepen the understanding of the molecular regulatory mechanism of lncRNAs in neurological development.

## 2 Materials and methods

### 2.1 Mouse models

The C57BL/6 mouse strain was used in this study. Six transgenic mice (experimental group) were provided by Cyagen Bioscience Inc. (Guangzhou, China). Eleven wild-type mice were provided by the Guangdong Medical Laboratory Animal Center (Guangzhou, China) and analyzed in parallel with the transgenic mice as a control group. All mice were placed in a specific pathogen-free cage and subjected to a 12 h light–dark cycle in an approved facility.

The transgenic mice were produced by cloning the human *HAR1* gene into an EF-1α promoter vector. The pRP (Exp)-EF1A vector (Cyagen Biosciences, Guangzhou, China) was used to construct the transgenic mice. The sequence of the target *HAR1* gene was: ATG​AAA​CGG​AGG​AGA​CGT​TAC​AGC​AAC​GTG​TCA​GCT​GAA​ATG​ATG​GGC​GTA​GAC​GCA​CGT​CAG​CGG​CGG​AAA​TGG​TTT​CTA​TCA​AAA​TGA​AAG​TGT​TTA​GAG​ATT​TTC​CTC​AAG​TTT​CA. The combined sequence of the *HAR1* gene and EF-1α promoter vector was named H119, with a length of 252 bp, and was randomly inserted into genomes.

All animal experiments were carried out according to the Experimental Animal Center of the Guangzhou Medical University’s guidelines and approved by the Guangzhou Medical University’s Ethics Committee (approval number: 2020-121).

### 2.2 Morris water maze (MWM) test

The experimental and control groups were trained in a round open pool (diameter: 1.7 m; depth: 0.3 m) at 23°C ± 1°C (Vorhees and Williams, 2006; Yu et al., 2018). The maze was divided into four equal quadrants (Pollard et al., 2006a; Pollard et al., 2006b; Kostka et al., 2012; Hubisz and Pollard, 2014) by specifying two orthogonal axes, the ends of which were marked as four base points: north (N), south (S), east (E), and west (W). A camera (FDR-AX700, Sony, Japan) was suspended from the middle of the ceiling to record the movements of the rats. The space acquisition task was tested four times a day, with an interval of 15 s, for five consecutive days.

The escape platform (diameter: 0.1 m) was approximately 1 cm below the water surface, located at the center of quadrant 2 (target quadrant). The mice were gently released from various starting points into the water and were allowed to seek the hidden platform for 60 s. Mice were manually guided to the target platform if they failed to find it within 60 s. After a 6-day delay from the last space acquisition training day, exploratory trials were conducted to assess the long-term memory of the mice (Yu et al., 2018). Tracking software (EthoVision XT 10, Noldus Information Technology, Leesburg, VA, United States) was used to analyze the results, including the escape latency in the spatial acquisition days and the latency to the target platform (probe time) during the probe trial.

### 2.3 Step-down test

The one-trial step-down test was performed to assess inhibitory avoidance and long-term memory, including 5-min training and a 5-min test after 24 h. The size of the chamber was approximately 0.18 (height) × 0.12 (width) × 0.12 (depth) m. The floorboard was an electrified grid composed of 1-mm copper bars in parallel, with a spacing of 0.05 m, and there was also a high-rising rubber platform (diameter: 0.24 m) in one corner of the chamber. Mice were placed on the platform with their noses directed toward the bottom corner. In the training session, when the mice stepped on the grid, they would receive an immediate shock (36V, AC), and as a result would instinctively jump up to the high-rising platform to avoid the electric shock. The time taken for the mice to jump from the high-rising platform to the grid bottom (step-down latency) and the number of times the mice jumped from the high-rising platform during the training phase (error counts) were recorded. In the experimental phase, the same procedure was conducted. The equipment was carefully cleaned after each test session to reduce the possibility of odor interference. After the step-down test, mice were euthanized by cervical dislocation, and their brain samples were subsequently collected (Yu et al., 2017).

### 2.4 RNA extraction

The TRIzol method (Invitrogen) was used to extract total RNA from the brain tissue of the experimental group mice and the control group mice. The Agilent 2100 Bioanalyzer was used to assess the concentration and quality of RNA.

### 2.5 Polymerase chain reaction (PCR) assay

Transgenic mice were screened by PCR assay. The internal control PCR targeted the endogenous mouse beta-actin *(Actb*) locus. The *HAR1* gene was amplified using primer F (5′-TAT​GCG​ATG​GAG​TTT​CCC​CAC​A-3′) and primer R (5′-GTC​TAC​GCC​CAT​CAT​TTC​AGC-3′), and the *Actb* gene was amplified using primer F (5′-ACT​CCA​AGG​CCA​CTT​ATC​ACC-3′) and primer R (5′-ATT​GTT​ACC​AAC​TGG​GAC​GAC​A-3′). PCR was carried out over 38 cycles in 25-µL tubes under standard conditions. The Taq DNA polymerase used here was TaKaRa R004A, and the control used in PCR genotyping was wild-type control: 400 ng, 25 µL of mouse genomic DNA.

The products were separated using 2% agarose gel electrophoresis. The gel was left to run for 90 min with an 80 V/70 A current. A UV transilluminator was used to visualize the products on the gel after red staining.

### 2.6 Sequencing and cDNA library construction

The TruSeq RNA Sample Preparation Kit (RS-200-0012, Illumina) was used to construct the cDNA library. The sequencing datasets were acquired using an Illumina NovaSeq 6000, and the datasets of the six experiment groups were named as follows: 4-6_HL3FVCCXY_L4, 5-5_HL3FVCCXY_L4, 6-4_HL3FVCCXY_L4, 7-1_HL3FVCCXY_L4, 8-7N_HL3FVCCXY_L4, and 9-3_HL3FVCCXY_L4.

The datasets of the 11 control groups were named as follows: 3-1N_HL3FVCCXY_L8, 3-9N_HL3FVCCXY_L8, 4-3N_HL3FVCCXY_L8, 4-4_HL3FVCCXY_L2, 4-9N_HL3FVCCXY_L8, 5-1_HL57YCCXY_L6, 5-4N_HL3FVCCXY_L4, 6-10_HL3FVCCXY_L4, 7-5N_HL3FVCCXY_L8, 7-6N_HL3FVCCXY_L8, and 8-5N_HL3FVCCXY_L4.

### 2.7 Differential gene expression analysis of the RNA-seq data

After obtaining the raw data, we performed quality control (QC) to remove adapters, N-terminus, and low-quality reads. FastQC (v 0.11.5) was used for QC with the parameters -a = given adapter sequence, --output = output path, and -m = 40 and others using default parameters. Trimmed reads sample data were aligned to reference sequences using Bowtie2 software (v2.2.6) with the following parameters: --read-edit- dist 3, -r 70, --library-type fr-unstranded, and others using default parameters. HTSeq-Count (0.6.1p1) was used for the expression quantification with the following parameters: --stranded = no, --format = bam, --- mode = intersection-nonempty, and others using default parameters. The DEGs between the experimental and control groups were screened using the DESeq2 package (1.12.4), with the following parameters: log2 (foldchange) >1, Padj <0.05. Here, Padj means the p-adjustment corrected by the Benjamini and Hochberg method after multiple test adjustments.

### 2.8 GO and KEGG pathway enrichment analyses

The DEGs were analyzed by cluster analysis, pathway significant enrichment analysis (Padj <0.05), and Gene Ontology (GO) functional enrichment analysis (Padj <0.05). GO enrichment analyses were based on the Wallenius non-central hypergeometric distribution. GO and KEGG enrichment analyses were conducted in the KOBAS website (http://kobas.cbi.pku.edu.cn). The database used in this research was the mouse full genome (UCSC version mm10).

### 2.9 Validation of differentially expressed genes

Ten DEGs involved in brain development were selected to validate the RNA-Seq results by quantitative real-time PCR (qRT-PCR). Total RNAs were first separated from the experimental and control group samples. The total RNAs were treated with DNase and were used to validate the RNA-seq results. After DNase treatment, 1.5 μg of DNase-treated RNA was reverse-transcribed to cDNA with oligo (dT) 15 using M-MLV reverse transcriptase (Life Technologies) in a 20 μL total reaction volume. Following the reverse transcription reaction, 1.5 μL of the reaction mixture was used to run a qPCR program of 46 cycles consisting of melting (30 s at 95°C), annealing (30 s at 60°C), and extension (30 s at 74°C). The 25 μL reaction mixture contained 1.5 PCR buffer (Mg^2+^ Plus), 1 μm of forward primer, 1 μm of reverse primer (Supplementary Table S1), 200 μm of each dNTP, and Roche SYBR-Green master mix in a LightCycler 480 Real-Time PCR System (Roche Applied Science). Data were analyzed by using the 2^**–∆∆Ct**^ method. Glyceraldehyde 3-phosphate dehydrogenase (*GADPH*) was used as the reference gene. All other results were shown as the fold change relative to the *GADPH* control.

## 3 Results

### 3.1 MWM and step-down passive avoidance tests

**FIGURE 1:**
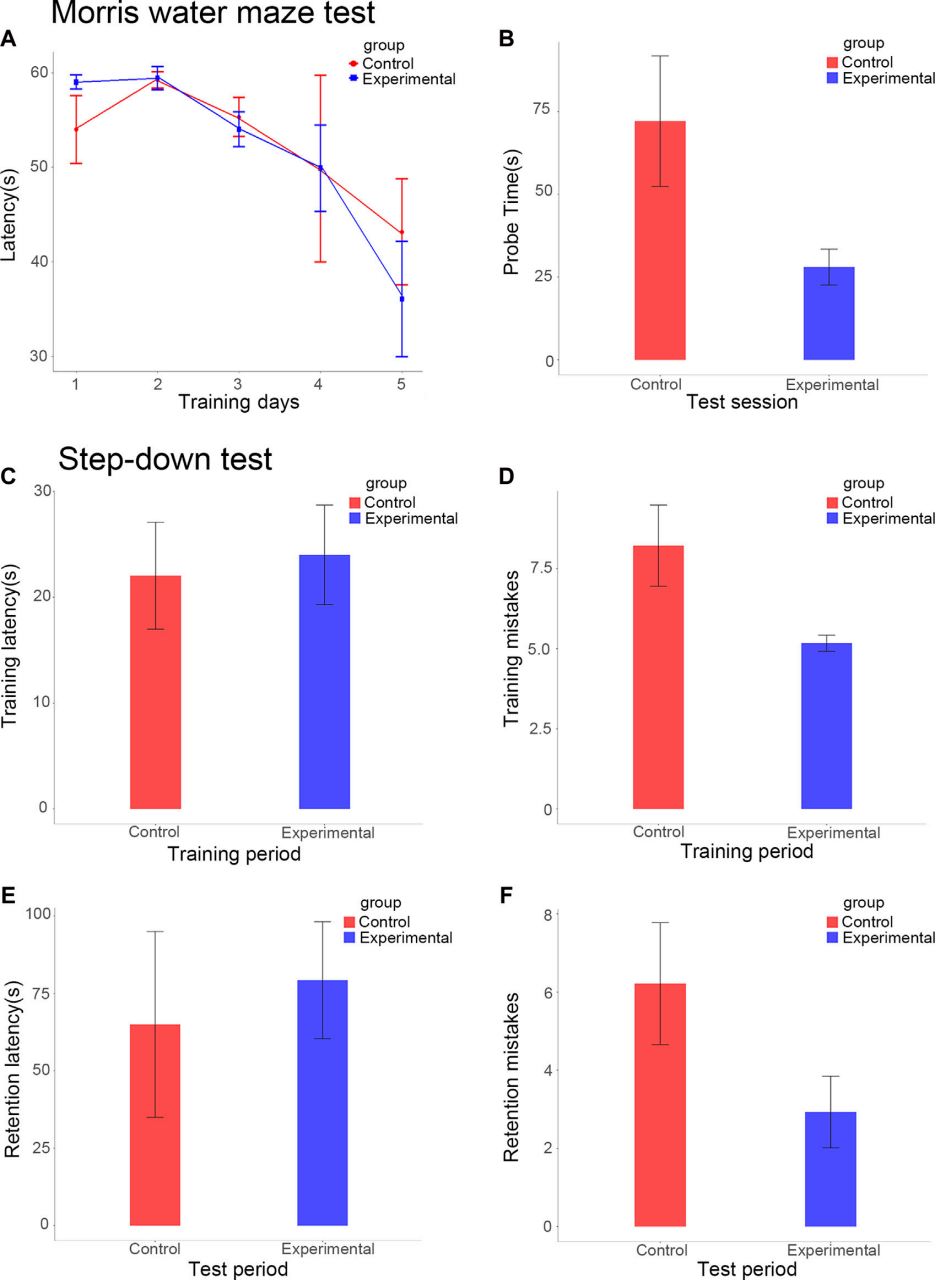
Escape latencies of the experimental and control groups over five consecutive training days **(A)**. Escape latencies of the experimental and control groups in the test session **(B)**. Step-down latency of the experimental and control groups during training **(C)** and test sessions **(E)**. Error counts in the experimental and control groups during the training **(D)** and test sessions **(F)**.

Our study used the MWM test to estimate spatial learning and long-term memory. During the space acquisition days, the time the mice took to find the hidden target platform (the escape latency) was measured. The escape latency was reduced during 5 days of training for the experimental group; the fifth day (36.0 ± 6.1 s) had about 61% of the value of the first day (59.0 ± 0.7 s). During the fifth day, the escape latency of the control group (43.1 ± 5.6 s) still accounted for about 80% of the value of the first day (54.0 ± 3.6 s) (Figure 1A). After a 6-day delay from the training course, a probe test was conducted to assess the long-term memory of the mice. In terms of the probe test, it took an average of 28.0 ± 5.4 s for the experimental group to reach the platform position for the first time, compared to 72.1 ± 19.7 s for the control group (*p* < 0.001; Figure 1B). Therefore, the duration of the probe test for the experimental group was significantly reduced compared to the control group.

This study measured the step-down latency and error count to assess memory retention (Figures 1C–F). During the training period, the latencies of the experimental and control groups were 24.0 ± 4.7 s and 22.0 ± 5.1 s respectively, this difference between the groups was not statistically significant (Figure 1C). During the test stage, the step-down latency of the experimental group increased by 79.2 ± 18.9 s, significantly more than that of the control group, which increased by 65.0 ± 30.0 s (*p* < 0.01; Figure 1E). The mice in the experimental group showed an overall smarter performance, with fewer errors in the training period (5.2 ± 0.3 vs 8.2 ± 1.3, *p* < 0.01; Figure 1D). During the test period, the error counts of the experimental group (2.9 ± 0.9) were significantly lower than those of the control group (6.2 ± 1.6, *p* < 0.01; Figure 1F).

The results of the MWM and step-down passive avoidance tests showed that *HAR1A* overexpression significantly improved the performance of both short- and long-term memory, spatial learning, and cognitive ability of mice.

### 3.2 Validation of transgenic mice

**FIGURE 2:**
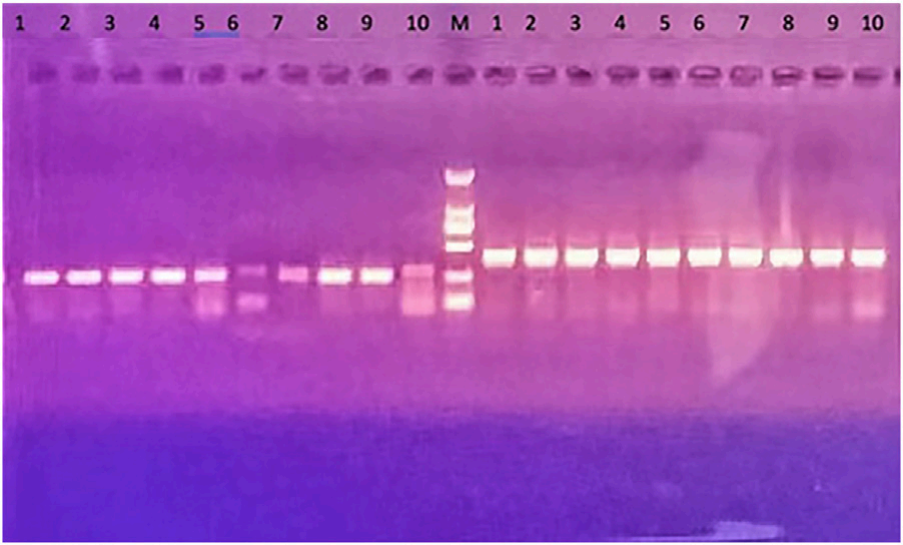
Results of PCR assay of transgenic mice. *HAR1* gene is on the left of the marker (M): transgene PCR products size: 252 bp; 6 strong positive: 1–4 and 8–9; 5–7 are general weak positive; No. 10 on the left is the control and is negative. The control gene is on the right side of the marker (M): internal control PCR product size: 413 bp; No. 10 is the control group, and its PCR product size: 413 bp.

The quality of transgenic mice was screened by PCR assay, which resulted in six strong positive results, 1–4 and 8–9 (Figure 2). This meant that the six mice successfully overexpressed *HAR1*. Their brain samples were then selected for follow-up experiments.

### 3.3 Review of the RNA-seq datasets

The QC results showed that the base composition was satisfied in all 17 samples. After excluding the adapter sequences and the low-quality reads, the lowest clean reading obtained from the 17 groups was 72,540,330. The number of mapped reads for each sample was more than 65 million, sufficient to provide valid data for further analysis. The map rates (mapped reads/raw reads) were all above 88% (Supplementary Table S2), suggesting that all the constructed libraries have excellent quality.

### 3.4 Analysis of DEGs between the experimental and control groups

**FIGURE 3:**
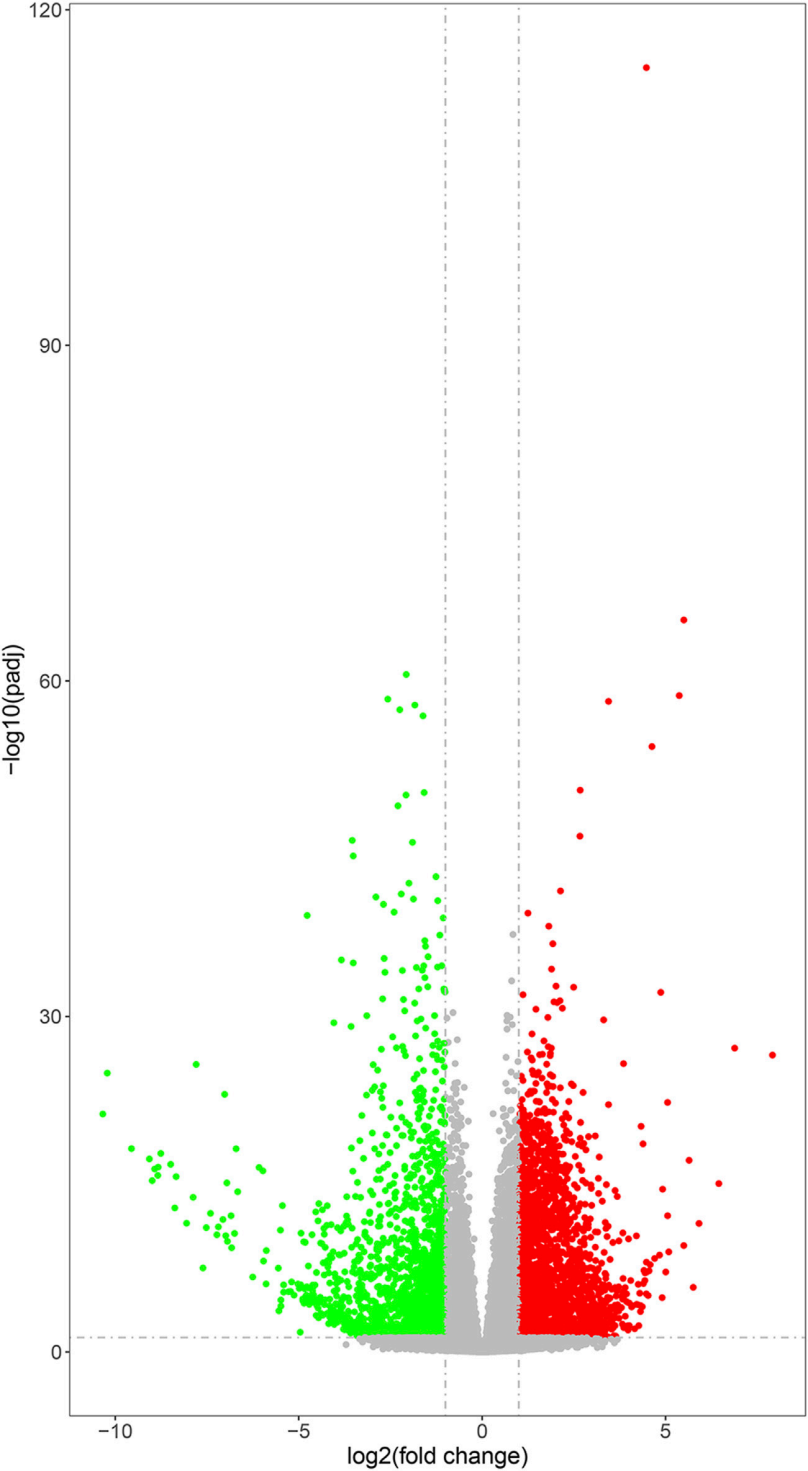
Volcano plots of DEGs. The gray points represent genes with relatively unchanged expression; the green points represent the downregulated genes; and the red points represent upregulated genes. *P*-value was adjusted using q-value and q-value < 0.005, and log2 (fold change) > 1 was set as the threshold for significant differential expression.

Differential gene expression analysis was performed to identify DEGs between the experimental and control groups. A total of 17,897 DEGs (fold change >1 and *p* < 0.05) were detected between the experimental and control groups, of which 7,933 genes were upregulated and 9,964 genes were downregulated (Figure 3). This result indicated the marked alterations in gene expression between the experimental and control groups.

### 3.5 GO functional enrichment analysis

**FIGURE 4:**
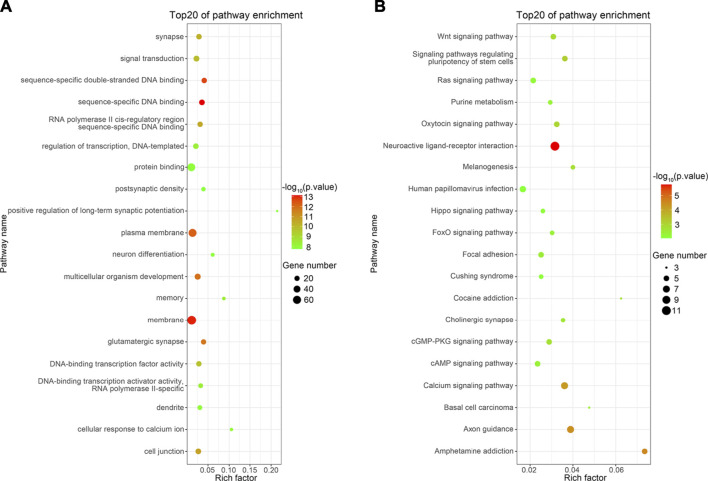
Top 20 functions enriched in GO analysis **(A)** and top 20 pathways enriched in KEGG analysis **(B)**. The Rich factor represents the enrichment degree of the terms, and the *y*-axis shows the names of the enriched terms. The area of the node represents the number of genes. The *p*-value is represented by a color scale. The statistical significance increased from green (relatively lower significance) to red (relatively higher significance).

GO enrichment analysis was performed to identify the primary functions of the DEGs. Ultimately, 1,361 important GO terms were obtained, and subsequently, the top 20 GO terms for functional enrichment were selected to configure a bubble chart (Figure 4A). “Positive regulation of long-term synaptic potentiation” and “Cellular response to calcium ions” exhibited the top two highest rich factor. Furthermore, DEGs were enriched in glutamatergic synapses, synapses, memory, and neuron differentiation, which are associated with neural development.

Table 1 presents the GO results related to brain development. Neuron differentiation, Dentate gyrus development, Nervous system development, Cerebral cortex neuron differentiation, Cerebral cortex development, Cerebral cortex development and Neurogenesis were all significant. They are mainly related to the directional differentiation of neural stem cells and the formation of spatial brain structures.

**TABLE 1:** Results of the GO analysis related to brain development.

Id	Description	*p*-value	Gene ID
GO:0098978	Glutamatergic synapse	1.74692E-12	*Stx1a*, *Akap5*, *Cnih3*, *Lrrc7*, *Synpo*, *Nrgn*, *Kalrn*, *Chrm4*, *Mal2*, *Camk2a*, *Chrm1*, *Drd1*, *Grin2b*, *Ptk2b*, *Arc*, *Cacng3*, *Camkv*, *Ngef*, *Homer2*, and *Shisa7*
GO:0045202	Synapse	5.27484E-11	*Cnih3*, *Synpo*, *Kcnj4*, *Camk2a*, *Ddn*, *Pdzrn3*, *Stx1a*, *Nrgn*, *Lrrc7*, *Syt5*, *Drd1*, *Grin2b*, *Shisa7*, *Homer2*, *Igfn1*, *Akap5*, *Kctd16*, *Chrm4*, *Chrm1*, *Dlgap2*, *Arc*, and *Grm2*
GO:0007613	Memory	4.47961E-09	*Kalrn*, *Plk2*, *Rin1*, *Drd1*, *Grin2b*, *Adrb1*, *Shisa7*, *Pak6*, and *Csmd1*
GO:1900273	Positive regulation of long-term synaptic potentiation	1.46644E-08	*Akap5*, *Nrgn*, *Kalrn*, *Drd1*, *Adrb1*, and *Shisa7*
GO:0030182	Neuron differentiation	1.57162E-08	*Mef2c*, *Lhx2*, *Lhx6*, *Wnt10a*, *Wnt1*, *Emx2*, *Tbr1*, *Emx1*, and *Wnt9a*
GO:0014069	Postsynaptic density	1.66394E-08	*Stx1a*, *Akap5*, *Lrrc7*, *Nrgn*, *Kalrn*, *Chrm4*, *Bcl11a*, *Camk2a*, *Chrm1*, *Grin2b*, *Ptk2b*, *Shisa7*, and *Homer2*
GO:0043197	Dendritic spine	4.20E-08	*Grin2b*, *Synpo*, *Akap5*, *Lrrc7*, *Camk2a*, *Drd1*, *Dlgap2*, *Ptk2b*, *Arc*, and *Arhgap33*
GO:0021542	Dentate gyrus development	8.08E-08	*Emx2*, *Neurod6*, *Nr2e1*, *Fezf2*, and *Drd1*
GO:0045211	Postsynaptic membrane	2.0757E-07	*Cnih3*, *Kctd16*, *Kcnj4*, *Chrm4*, *Chrm1*, *Grin2b*, *Nrgn*, *Arc*, *Ddn*, and *Shisa7*
GO:0043025	Neuronal cell body	5.06105E-07	*Camk2a*, *Akap5*, *Kcnj4*, *Kalrn*, *Pde1a*, *Chrm4*, *Rin1*, *Syt5*, *Cpne5*, *Drd1*, *Grin2b*, *Nrgn*, *Ptk2b*, *Enc1*, and *Homer2*
GO:0007399	Nervous system development	1.86339E-06	*Mef2c*, *Lhx2*, *Neurod6*, *Lhx6*, *Fezf2*, *Kalrn*, *Robo3*, *Ntn5*, *Enc1*, *Ngef*, and *Islr2*
GO:0021895	Cerebral cortex neuron differentiation	3.03972E-06	*Lhx6*, *Nr2e1*, *Fezf2*, and *Emx1*
GO:0021987	Cerebral cortex development	1.13404E-05	*Lhx2*, *Emx2*, *Foxg1*, *Nr2e1*, and *Emx1*
GO:0021902	Commitment of neuronal cell to a specific neuron type in the forebrain	1.32497E-05	*Satb2*, *Fezf2*, and *Tbr1*
GO:0048664	Neuron fate determination	1.32497E-05	*Foxg1*, *Wnt1*, and *Fezf2*
GO:0048168	Regulation of neuronal synaptic plasticity	1.35E-05	*Grin2b*, *Kalrn*, *Arc*, and *Camk2a*
GO:0000976	Transcription regulatory region sequence-specific DNA binding	2.21E-05	*Sp9*, *Grhl1*, *Fezf2*, *Egr2*, *Egr3*, *Egr1*, *Mef2c*, *Ovol2*, and *Dlx5*
GO:0048169	Regulation of long-term neuronal synaptic plasticity	2.52E-05	*Grin2b*, *Egr1*, *Synpo*, and *Drd1*
GO:0022008	Neurogenesis	2.96E-05	*Wnt1*, *Lhx2*, *Foxg1*, and *Ntn5*
GO:0099061	Integral component of the postsynaptic density membrane	3.60E-05	*Grin2b*, *Chrm4*, *Lrrc7*, *Chrm1*, and *Cacng3*

Considering that using multiple databases provides more reliable results, we performed a series of analyses on DEGs using GO, REAC (https://reactome.org/), and HPA databases (https://www.atlasantibodies.com/). As shown in Table 2, the *p*-values for the top 13 enrichment pathways were all below 4.7E-43, indicating a high significance. The neuron part pathway, synaptic signaling pathway, neuronal system pathway, and pre-synapse pathway were all significantly enriched.

**TABLE 2:** Results of GO, REAC, and HPA enrichment analyses.

	Enrichment analysis	GO enrichment pathway	*p*-value
1	GO:0097458	Neuron part	5.1E-81
2	GO:0045202	Synapse	1.8E-75
3	GO:0099536	Synaptic signaling	2.4E-75
4	GO:0099537	Trans-synaptic signaling	2.4E-75
5	GO:0098916	Anterograde trans-synaptic signaling	2.4E-75
6	GO:0007268	Chemical synaptic transmission	2.4E-75
7	GO:0044456	Synapse part	8.4E-73
8	REAC:112316	Neuronal system	5.4E-70
9	GO:0043005	Neuron projection	2.7E-59
10	REAC:112315	Transmission across chemical synapses	3.8E-45
11	HPA:007040_02	Cerebral cortex; neuropil	1.2E-43
12	GO:0097060	Synaptic membrane	2.5E-43
13	GO:0098793	Presynapse	4.7E-43

### 3.6 KEGG pathway analysis

KEGG pathway analysis was performed to investigate the pathways in which DEGs were significantly enriched. Apart from GO terms, 124 KEGG pathways were found to be related to the DEGs. The top 20 most significantly enriched pathways are shown in Figure 4B. The neuroactive ligand–receptor interaction pathway showed the greatest significance. Furthermore, DEGs were notably enriched in terms of axon guidance, calcium signaling, and cholinergic synapse, all related to neural development (Table 3) (Sanes and Zipursky, 2020).

**TABLE 3:** Results of KEGG analysis related to brain development.

ID	Description	*p*-value	GENE ID
mmu04080	Neuroactive ligand–receptor interaction	1.70E-06	*Cort*, *Mas1*, *Rxfp1*, *Chrm4*, *Chrm1*, *Drd1*, *Grin2b*, *Adrb1*, *Sstr4*, *Glp2r*, and *Grm2*
mmu04360	Axon guidance	3.90E-05	*Ngef*, *Robo3*, *Camk2a*, *Trpc6*, *Trpc4*, *Pak6*, and *Nov*
mmu04020	Calcium signaling pathway	6.16E-05	*Camk2a*, *Pde1a*, *Mylk3*, *Chrm1*, *Drd1*, *Adrb1*, and *Ptk2b*
mmu04725	Cholinergic synapse	2.62E-03	*Chrm4*, *Camk2a*, *Chrm1*, and *Kcnj4*

### 3.7 Validation of the DEGs using qRT-PCR

**FIGURE 5:**
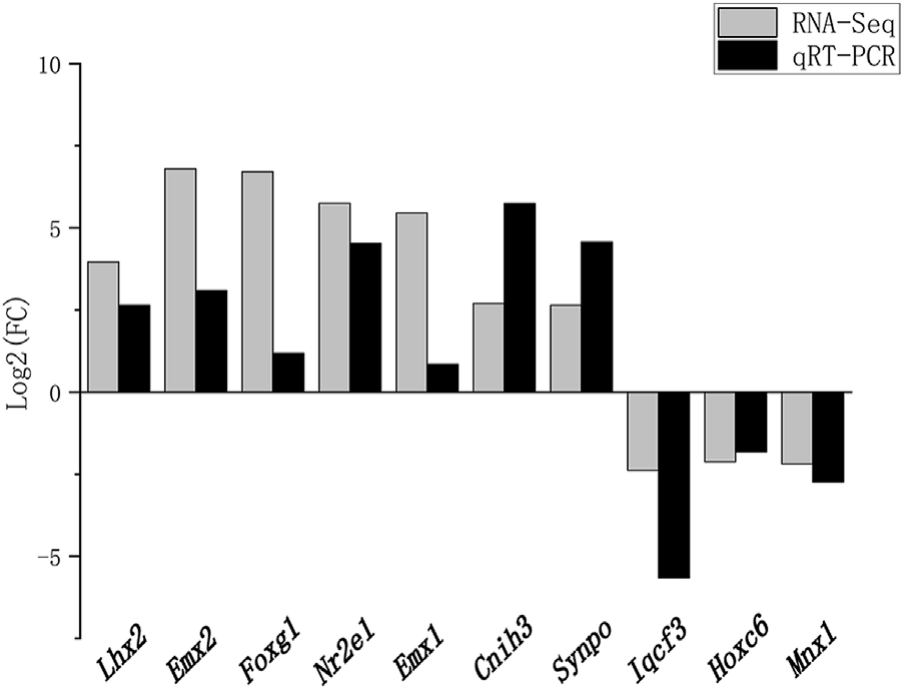
Validation of RNA-seq data using RT-qPCR. Fold changes in gene expression between the experimental and control groups detected by RNA-seq were compared with those measured by qRT-PCR. The mRNA levels were normalized to *GADPH* expression, and the expression fold changes (compared to the control sample) were calculated by using the 2^–∆∆Ct^ method. The x- and *y*-axes correspond to the genes and log2 (ratio of H/L) measured by RNA-seq and qPCR.

Ten DEGs involved in brain development were chosen for qRT-PCR confirmation. Consistent with the results of RNA-seq, *Lhx2*, *Emx2*, *Foxg1*, *Nr2e1*, *Emx1*, *Cnih3*, and *Synpo* were upregulated, and *Iqcf3*, *Hoxc6*, and *Mnx1* were downregulated (Figure 5).

## 4 Discussion

Previous studies have confirmed that HARs are related to the development and evolution of the human brain (Tolosa et al., 2008; Mosti and Silver, 2021). *HAR1* is a section of *HAR1A* that has been conserved through vertebrate evolution, although a difference of 18 mutations in its 118-bp sequence between humans and chimpanzees is present (Pollard et al., 2006a; Pollard et al., 2006b). *HAR1* is part of an overlapping cis-antisense pair of structured lncRNAs, *HAR1A* and *HAR1B*. The expression of lncRNA *HAR1B* in the human brain is lower than that of *HAR1A*. The expression pattern of *HAR1B* suggests that it may be expressed in later stages of brain development to downregulate *HAR1A* by antisense inhibition (Pollard et al., 2006a). The human mutations in the *HAR1* region result in the corresponding secondary structural changes in lncRNA *HAR1A*; however, research on the impact of this change is still limited. This study confirmed that *HAR1* overexpression led to a significant improvement in the cognition and memory of mice. The possible pathways and mechanisms were then explored through bioinformatics analysis.

### 4.1 Potential pathways of *HAR1A* affecting cortical development through CRs

LncRNA *HAR1A* is specifically expressed in CRs, which exist in the developing human neocortex during the gestational period of weeks 7–19 (Waters et al., 2021). CRs are crucial in the specification and migration of cortical neurons, suggesting that *HAR1A* plays an important role in neurogenesis (Causeret et al., 2021). The results of the GO analysis showed that Neuron differentiation, Dentate gyrus development, Nervous system development, Cerebral cortex neuron differentiation, Cerebral cortex development, Cerebral cortex development and Neurogenesis are all significant GO terms related to brain development. Five DEGs repeatedly appear in these GO items process: *Lhx2*, *Emx2*, *Foxg1*, *Nr2e1*, and *Emx1*.

*Foxg1* encodes a transcription repression factor essential in the regional subdivision for telencephalon formation and brain development (Hettige and Ernst, 2019). When *Foxg1* is expressed in cortical progenitor cells, this stops the production of CRs by directly inhibiting a default transcriptional network (Hanashima et al., 2004). *Foxg1*–*Lhx2* interactions instruct the termination of CR production (Godbole et al., 2018). CRs originate from the cortical hem (Griveau et al., 2010). Neurons in the ventral pallium and cortical hem in the medial pallium become subplate and CRs, respectively, after developing and migrating toward the neuroepithelium (Griveau et al., 2010; García-Moreno and Molnár, 2020). *Emx1* and *Emx2* are required to establish the Wnt-rich cortical hem domain (Dixit et al., 2011; von Frowein et al., 2006). Both *EMX1* and *EXM2* encode a homeobox-containing transcription factor and cooperate to promote the generation of CRs. *Nr2e1* participates in a feedback loop with the brain-specific microRNA-9 (miR-9), while mouse miR-9 targets *Foxg1* to control the generation of CRs in the medial pallium properly (Davila et al., 2014).

CRs generated early in the marginal zone of the cortex synthesize the glycoprotein reelin and secrete it into the peripheral intercellular stroma to regulate the morphology and biochemical maturation of radial glia cells (RGCs) (Lehrman et al., 2018; Junqueira Alves et al., 2021). RGC fibers act as scaffolding when newborn neurons migrate to their final destinations. Reelin is a stop signal for neuron migration, which decides the neurons’ orientation and positioning within their layers. However, abnormal function of CRs can lead to various neurological diseases, including Alzheimer’s disease, schizophrenia, lissencephaly, and temporal lobe epilepsy (Chen et al., 2017).

LncRNA *HAR1A* may affect the function of CRs by regulating the expression of *Lhx2*, *Emx2*, *Foxg1*, *Nr2e1*, and *Emx1*. *HAR1A* may affect cerebral cortex development in this way, although the specific mechanism of this requires further study.

### 4.2 LncRNA *HAR1A* may affect synaptic function and formation

The KEGG pathway analysis results showed that the neuroactive ligand–receptor interaction pathway was the most significant, and DEGs were notably enriched in axon guidance, and cholinergic synapses. The results of GO analysis showed that DEGs were enriched in glutamatergic synapses, synapses, memory, and the positive regulation of long-term synaptic potentiation. Multiple database analyses (GO, REAC, and HPA) of DEGs showed that the synaptic signaling pathway and pre-synapse pathway were highly significant.

Based on these results, it can be concluded that HAR1A holds an important role in neuroactive ligand–receptor interactions and synaptic and axon guidance, all of which are critical processes in the early development of the brain (Wang et al., 2021). Neurons migrate to their correct locations to make connections by emitting axons during the development of the brain (Ulloa et al., 2018). Multiple environmental signals control the migration of these neurons and axon growth. After axons reach their appropriate position, synapses will be formed to link neurons together (Gangatharan et al., 2018). Axonal branches are over-formed during the early development of the brain; then, specific neuronal connections are formed by removing the redundant synapses (Lehrman et al., 2018; Luck et al., 2019). Several ligand–receptor pairs are involved in these cellular events (Junqueira Alves et al., 2021).

Existing evidence has shown that HARs regulate neurodevelopmental processes that have diverged between humans and chimpanzees, such as synapse development (Won et al., 2019). The human prefrontal cortex (PFC) is enlarged compared to other portions of the brain and is related to the increasing neocortical volume in primates. Changes in the synaptic distribution in the primate PFC may be caused by differential expression and transcription patterns of specific genes (Pattabiraman et al., 2020). Based on our experimental results and the conclusions of existing studies, it can be speculated that *HAR1A* may affect the evolution of the human brain by regulating the expression of genes related to synaptic generation.

### 4.3 *HAR1A* regulates the crucial second signal calcium ion during cortical development

The GO analysis results showed that “cellular response to calcium ion” of the “biological process” exhibited the second highest rich factor value.

Calcium plays a crucial role in neurogenesis as an essential second messenger (Callens et al., 2021). Neural stem cells stay in the neocortex’s ventricular zone to produce progenitor cells, glial cells, and subsequently, neurons, which compose the entire adult brain (Sokpor et al., 2018). Multiple signals strictly regulate proliferation, migration, and differentiation during the establishment of the structure of the cerebral cortex, including cytosolic calcium ions. The regulation of Ca^2+^ is essential in the delicate process of cortical development.

## 5 Conclusion

Our study confirmed that *HAR1* overexpression improved the memory and cognitive abilities of transgenic C57BL/6 mice. We identified the *HAR1* regulatory network in the mice by RNA-Seq. The results showed that *HAR1* may affect brain development through CRs. Also, it is related to ligand–receptor interaction, axon guidance, and synapse formation, which are important processes in brain development and evolution. *HAR1* also played an important role in the regulation of the second signal calcium ion. These findings provide valuable insights into the molecular mechanisms of how *HAR1* affects brain development and the potential role of HARs in the evolution of the brain.

## Data Availability

The datasets presented in this study can be found in online repositories (https://www.ncbi.nlm.nih.gov/geo/query/acc.cgi?acc=GSE197554).

## Abbreviations

CRs, Cajal—Retzius neurons; RGCs, radial glia cells; DEGs, differentially expressed genes; lncRNA, long non-coding RNA; MWM test, Morris water maze test; TCGA, The Cancer Genome Atlas; GO analysis, Gene Ontology analysis; KEGG, Kyoto Encyclopedia of Genes and Genomes; qRT-PCR, quantitative real-time PCR.

## References

[B1] Callens M. Kraskovskaya N. Derevtsova K. Annaert W. Bultynck G. Bezprozvanny I. (2021). The role of Bcl-2 proteins in modulating neuronal Ca(2+) signaling in health and in Alzheimer's disease. Biochimica biophysica acta Mol. Cell Res. 1868 (6), 118997. Epub 2021/03/13PubMed PMID: 33711363; PubMed Central PMCID: PMCPMC8041352. 10.1016/j.bbamcr.2021.118997 PMC8041352 33711363

[B2] Cao Y. Zhu H. Tan J. Yin W. Zhou Q. Xin Z. (2021). Development of an immune-related LncRNA prognostic signature for glioma. Front. Genet. 12, 678436. Epub 2021/07/02PubMed PMID: 34194477; PubMed Central PMCID: PMCPMC8238205. 10.3389/fgene.2021.678436 34194477 PMC8238205

[B3] Causeret F. Moreau M. X. Pierani A. Blanquie O. (2021). The multiple facets of Cajal-Retzius neurons. Development 148 (11), dev199409. Epub 2021/05/29PubMed PMID: 34047341. 10.1242/dev.199409 34047341

[B4] Chen M. Wang J. Luo Y. Huang K. Shi X. Liu Y. (2018). Identify Down syndrome transcriptome associations using integrative analysis of microarray database and correlation-interaction network. Hum. genomics 12 (1), 2. Epub 2018/01/21PubMed PMID: 29351810; PubMed Central PMCID: PMCPMC5775600. 10.1186/s40246-018-0133-y 29351810 PMC5775600

[B5] Chen X. Yan C. C. Zhang X. You Z. H. (2017). Long non-coding RNAs and complex diseases: From experimental results to computational models. Briefings Bioinforma. 18 (4), 558–576. Epub 2016/06/28PubMed PMID: 27345524; PubMed Central PMCID: PMCPMC5862301. 10.1093/bib/bbw060 PMC5862301 27345524

[B6] Davila J. L. Goff L. A. Ricupero C. L. Camarillo C. Oni E. N. Swerdel M. R. (2014). A positive feedback mechanism that regulates expression of miR-9 during neurogenesis. PLoS One 9 (4), e94348. Epub 2014/04/10PubMed PMID: 24714615; PubMed Central PMCID: PMCPMC3979806. 10.1371/journal.pone.0094348 24714615 PMC3979806

[B7] Dixit R. Zimmer C. Waclaw R. R. Mattar P. Shaker T. Kovach C. (2011). *Ascl1* participates in Cajal-Retzius cell development in the neocortex. Cereb. cortex 21 (11), 2599–2611. Epub 2011/04/07PubMed PMID: 21467208. 10.1093/cercor/bhr046 21467208

[B8] Gangatharan G. Schneider-Maunoury S. Breau M. A. (2018). Role of mechanical cues in shaping neuronal morphology and connectivity. Biol. Cell 110 (6), 125–136. Epub 2018/04/27PubMed PMID: 29698566. 10.1111/boc.201800003 29698566

[B9] García-Moreno F. Molnár Z. (2020). Variations of telencephalic development that paved the way for neocortical evolution. Prog. Neurobiol. 194, 101865. Epub 2020/06/12PubMed PMID: 32526253; PubMed Central PMCID: PMCPMC7656292. 10.1016/j.pneurobio.2020.101865 32526253 PMC7656292

[B10] Godbole G. Shetty A. S. Roy A. D'Souza L. Chen B. Miyoshi G. (2018). Hierarchical genetic interactions between *FOXG1* and *LHX2* regulate the formation of the cortical hem in the developing telencephalon. Development 145 (1), dev154583. Epub 2017/12/13PubMed PMID: 29229772; PubMed Central PMCID: PMCPMC5825872. 10.1242/dev.154583 29229772 PMC5825872

[B11] Griveau A. Borello U. Causeret F. Tissir F. Boggetto N. Karaz S. (2010). A novel role for Dbx1-derived Cajal-Retzius cells in early regionalization of the cerebral cortical neuroepithelium. PLoS Biol. 8 (7), e1000440. Epub 2010/07/30PubMed PMID: 20668538; PubMed Central PMCID: PMCPMC2910656. 10.1371/journal.pbio.1000440 20668538 PMC2910656

[B12] Hanashima C. Li S. C. Shen L. Lai E. Fishell G. (2004). *Foxg1* suppresses early cortical cell fate. Sci. (New York, NY) 303 (5654), 56–59. Epub 2004/01/06PubMed PMID: 14704420. 10.1126/science.1090674 14704420

[B13] Hettige N. C. Ernst C. (2019). *FOXG1* dose in brain development. Front. Pediatr. 7, 482. Epub 2019/12/12PubMed PMID: 31824897; PubMed Central PMCID: PMCPMC6882862. 10.3389/fped.2019.00482 31824897 PMC6882862

[B14] Hubisz M. J. Pollard K. S. (2014). Exploring the Genesis and functions of Human Accelerated Regions sheds light on their role in human evolution. Curr. Opin. Genet. Dev. 29, 15–21. Epub 2014/08/27PubMed PMID: 25156517. 10.1016/j.gde.2014.07.005 25156517

[B15] Junqueira Alves C. Silva Ladeira J. Hannah T. Pedroso Dias R. J. Zabala Capriles P. V. Yotoko K. (2021). Evolution and diversity of semaphorins and plexins in choanoflagellates. Genome Biol. Evol. 13 (3), evab035. Epub 2021/02/25PubMed PMID: 33624753; PubMed Central PMCID: PMCPMC8011033. 10.1093/gbe/evab035 33624753 PMC8011033

[B16] Kostka D. Hubisz M. J. Siepel A. Pollard K. S. (2012). The role of GC-biased gene conversion in shaping the fastest evolving regions of the human genome. Mol. Biol. Evol. 29 (3), 1047–1057. Epub 2011/11/15PubMed PMID: 22075116; PubMed Central PMCID: PMCPMC3278478. 10.1093/molbev/msr279 22075116 PMC3278478

[B17] Lee C. P. Ko A. M. Nithiyanantham S. Lai C. H. Ko Y. C. (2021). Long non-coding RNA *HAR1A* regulates oral cancer progression through the alpha-kinase 1, bromodomain 7, and myosin IIA axis. J. Mol. Med. (Berlin, Ger. 99 (9), 1323–1334. Epub 2021/06/08PubMed PMID: 34097087. 10.1007/s00109-021-02095-x 34097087

[B18] Lehrman E. K. Wilton D. K. Litvina E. Y. Welsh C. A. Chang S. T. Frouin A. (2018). CD47 protects synapses from excess microglia-mediated pruning during development. Neuron 100 (1), 120–134.e6. e6. Epub 2018/10/12PubMed PMID: 30308165; PubMed Central PMCID: PMCPMC6314207. 10.1016/j.neuron.2018.09.017 30308165 PMC6314207

[B19] Luck R. Urban S. Karakatsani A. Harde E. Sambandan S. Nicholson L. (2019). *VEGF/VEGFR2* signaling regulates hippocampal axon branching during development. eLife 8, e49818. Epub 2019/12/24PubMed PMID: 31868583; PubMed Central PMCID: PMCPMC6927742. 10.7554/eLife.49818 31868583 PMC6927742

[B20] Mosti F. Silver D. L. (2021). Uncovering the HARbingers of human brain evolution. Neuron 109 (20), 3231–3233. Epub 2021/10/22PubMed PMID: 34672980. 10.1016/j.neuron.2021.09.022 34672980

[B21] Pattabiraman K. Muchnik S. K. Sestan N. (2020). The evolution of the human brain and disease susceptibility. Curr. Opin. Genet. Dev. 65, 91–97. Epub 2020/07/07PubMed PMID: 32629339. 10.1016/j.gde.2020.05.004 32629339

[B22] Pollard K. S. Salama S. R. King B. Kern A. D. Dreszer T. Katzman S. (2006). Forces shaping the fastest evolving regions in the human genome. PLoS Genet. 2 (10), e168. Epub 2006/10/17PubMed PMID: 17040131; PubMed Central PMCID: PMCPMC1599772. 10.1371/journal.pgen.0020168 17040131 PMC1599772

[B23] Pollard K. S. Salama S. R. Lambert N. Lambot M. A. Coppens S. Pedersen J. S. (2006). An RNA gene expressed during cortical development evolved rapidly in humans. Nature 443 (7108), 167–172. Epub 2006/08/18PubMed PMID: 16915236. 10.1038/nature05113 16915236

[B24] Sanes J. R. Zipursky S. L. (2020). Synaptic specificity, recognition molecules, and assembly of neural circuits. Cell 181 (6), 1434–1435. Epub 2020/06/13PubMed PMID: 32531247. 10.1016/j.cell.2020.05.046 32531247

[B25] Sokpor G. Castro-Hernandez R. Rosenbusch J. Staiger J. F. Tuoc T. (2018). ATP-dependent chromatin remodeling during cortical neurogenesis. Front. Neurosci. 12, 226. Epub 2018/04/25PubMed PMID: 29686607; PubMed Central PMCID: PMCPMC5900035. 10.3389/fnins.2018.00226 29686607 PMC5900035

[B26] Tolosa A. Sanjuán J. Leal C. Costas J. Moltó M. D. de Frutos R. (2008). Rapid evolving RNA gene *HAR1A* and schizophrenia. Schizophrenia Res. 99 (1-3), 370–372. Epub 2007/12/07PubMed PMID: 18054202. 10.1016/j.schres.2007.10.011 18054202

[B27] Ulloa F. Cotrufo T. Ricolo D. Soriano E. Araújo S. J. (2018). *SNARE* complex in axonal guidance and neuroregeneration. Neural Regen. Res. 13 (3), 386–392. Epub 2018/04/07PubMed PMID: 29623913; PubMed Central PMCID: PMCPMC5900491. 10.4103/1673-5374.228710 29623913 PMC5900491

[B28] von Frowein J. Wizenmann A. Götz M. (2006). The transcription factors *Emx1* and *Emx2* suppress choroid plexus development and promote neuroepithelial cell fate. Dev. Biol. 296 (1), 239–252. Epub 2006/06/24PubMed PMID: 16793035. 10.1016/j.ydbio.2006.04.461 16793035

[B29] Vorhees C. V. Williams M. T. (2006). Morris water maze: Procedures for assessing spatial and related forms of learning and memory. Nat. Protoc. 1 (2), 848–858. Epub 2007/04/05PubMed PMID: 17406317; PubMed Central PMCID: PMCPMC2895266. 10.1038/nprot.2006.116 17406317 PMC2895266

[B30] Wang X. Li Z. Zhu Y. Yan J. Liu H. Huang G. (2021). Maternal folic acid impacts DNA methylation profile in male rat offspring implicated in neurodevelopment and learning/memory abilities. Genes and Nutr. 16 (1), 1. Epub 2021/01/13PubMed PMID: 33430764; PubMed Central PMCID: PMCPMC7802276. 10.1186/s12263-020-00681-1 PMC7802276 33430764

[B31] Waters E. Pucci P. Hirst M. Chapman S. Wang Y. Crea F. (2021). *HAR1*: An insight into lncRNA genetic evolution. Epigenomics 13 (22), 1831–1843. Epub 2021/10/23PubMed PMID: 34676772. 10.2217/epi-2021-0069 34676772

[B32] Wei M. Huang J. Li G. W. Jiang B. Cheng H. Liu X. (2021). Axon-enriched lincRNA *ALAE* is required for axon elongation via regulation of local mRNA translation. Cell Rep. 35 (5), 109053. Epub 2021/05/06PubMed PMID: 33951423. 10.1016/j.celrep.2021.109053 33951423

[B33] Won H. Huang J. Opland C. K. Hartl C. L. Geschwind D. H. (2019). Human evolved regulatory elements modulate genes involved in cortical expansion and neurodevelopmental disease susceptibility. Nat. Commun. 10 (1), 2396. Epub 2019/06/05PubMed PMID: 31160561; PubMed Central PMCID: PMCPMC6546784. 10.1038/s41467-019-10248-3 31160561 PMC6546784

[B34] Yu H. Lin X. Wang D. Zhang Z. Guo Y. Ren X. (2018). Mitochondrial molecular abnormalities revealed by proteomic analysis of hippocampal organelles of mice triple transgenic for alzheimer disease. Front. Mol. Neurosci. 11, 74. Epub 2018/03/30PubMed PMID: 29593495; PubMed Central PMCID: PMCPMC5854685. 10.3389/fnmol.2018.00074 29593495 PMC5854685

[B35] Yu J. Mu J. Guo Q. Yang L. Zhang J. Liu Z. (2017). Transcriptomic profile analysis of mouse neural tube development by RNA-Seq. IUBMB life 69 (9), 706–719. Epub 2017/07/12PubMed PMID: 28691208. 10.1002/iub.1653 28691208

