# Supplementary material for: Effects of HAR1 on cognitive function in mice and the regulatory network of HAR1 determined by RNA sequencing and applied bioinformatics analysis
Source: Front Genet. 2023 Mar 8;14:947144. doi: 10.3389/fgene.2023.947144 (PMC10030831; doi:10.3389/fgene.2023.947144)
Supplement: Supplementary file 1 [file Table1.docx]

Spplement Table 1: Primer pairs for qRT-PCR of DEGs involved in brain development.

| Gene symbol | Gene ID | Primer (5' → 3') |
| --- | --- | --- |
| *Lhx2* | NM_010710 | F: GATGCCAAGGACTTGAAGCAGC  R: TTCCTGCCGTAAAAGGTTGCGC |
| *Emx2* | [NM_010132.2](https://www.ncbi.nlm.nih.gov/nuccore/NM_010132.2) | F: TCCAAGGGAACGACACAAGT  R: CCGGTTAATGTGGTGTGTCC |
| *Foxg1* | NM_001160112.1 | F: CCCTGCCCTGTGAGTCTTTA  R: GGTTGGAAGAAGACCCCTGA |
| *Nr2e1* | NM_152229 | F: CGCCACTGAATTTGCCTGTCTG  R: CCTCATCTTGGAGAGCGGCAAT |
| *Emx1* | NM_004097 | F: GCCTTCGAGAAGAACCACTACG  R: CGGTTCTGGAACCACACCTTCA |
| *Cnih3* | NM_001160211 | F: ACCCTCAACTCTGTCTCTGC  R: CGTCAAAGGCGATTATGTGC |
| *Synpo* | NM_007286 | F: AGGAGGTGAGATGCAGCACACT  R: TAGGGTGTTGGGCTGGATGTCA |
| *Iqcf3* | NM_001368648.1 | F: GCGCTACAGACTGAGAAACC  R: TGCCTCTTCCTCAATGACGT |
| *Hoxc6* | NM_010465 | F: TTACCCCTGGATGCAGCGAATG  R: CCGAGTTAGGTAGCGGTTGAAG |
| *Mnx1* | NM_019944.2 | F: CATGATCCTGCCCAAGATGC  R: ATCTTCACCTGAGTCTCGGT |
| *GADPH* | NM_001256799 | F: TGGAAGGACTCATGACCACA  R: ATGATGTTCTGGAGAGCCCC |
