# Supplementary material for: Effects of HAR1 on cognitive function in mice and the regulatory network of HAR1 determined by RNA sequencing and applied bioinformatics analysis
Source: Front Genet. 2023 Mar 8;14:947144. doi: 10.3389/fgene.2023.947144 (PMC10030831; doi:10.3389/fgene.2023.947144)
Supplement: Supplementary file 2 [file Table2.doc]

Supplement Table 2. Sequencing reads number of different groups of mice

| **sample_id** | **group** | **raw_reads** | **clean_reads** | **mapped_reads** |
| --- | --- | --- | --- | --- |
| **3-1N_HL3FVCCXY_L8** | control | 79312928 | 79253222 | 70219241 |
| **3-9N_HL3FVCCXY_L8** | control | 85405460 | 85316740 | 76309652 |
| **4-3N_HL3FVCCXY_L8** | control | 72612004 | 72540330 | 65026158 |
| **4-4_HL3FVCCXY_L2** | control | 80117816 | 80026942 | 71216056 |
| **4-6_HL3FVCCXY_L4** | case | 80192986 | 80124336 | 72195909 |
| **4-9N_HL3FVCCXY_L8** | control | 80282660 | 80207448 | 71801854 |
| **5-1_HL57YCCXY_L6** | control | 91129066 | 91083962 | 81399419 |
| **5-4N_HL3FVCCXY_L4** | control | 91384118 | 91307294 | 81740179 |
| **5-5_HL3FVCCXY_L4** | case | 89480536 | 89402044 | 80713062 |
| **6-10_HL3FVCCXY_L4** | control | 83319872 | 83242190 | 75149506 |
| **6-4_HL3FVCCXY_L4** | case | 82980490 | 82898530 | 74828041 |
| **7-1_HL3FVCCXY_L4** | case | 93213296 | 93125238 | 83373952 |
| **7-5N_HL3FVCCXY_L8** | control | 87108060 | 87026102 | 76998314 |
| **7-6N_HL3FVCCXY_L8** | control | 87314292 | 87234908 | 77238419 |
| **8-5N_HL3FVCCXY_L4** | control | 78169916 | 78111572 | 70444218 |
| **8-7N_HL3FVCCXY_L4** | case | 90977174 | 90899290 | 81926184 |
| **9-3_HL3FVCCXY_L4** | case | 77339916 | 77272796 | 68799994 |
